# E-cigarette use and cigarette smoking initiation studies: A word of caution

**DOI:** 10.18332/tpc/112655

**Published:** 2019-11-06

**Authors:** Red Thaddeus D. Miguel, Isabella Steffensen

**Affiliations:** 1 Thera Business Inc., Ottawa, Canada

**Keywords:** e-cigarette, meta-analysis, smoking initiation, confounders, covariates, tobacco

**Dear Editor,**

**Figure 1:**
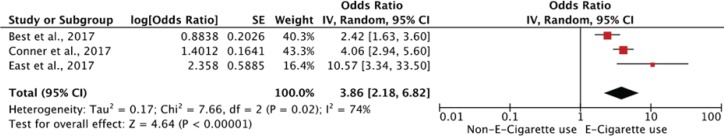
Meta-analysis based on adjusted odds ratios

In a recent review, published in Tobacco Prevention & Cessation, entitled: ‘Is adolescent e-cigarette use associated with smoking in the United Kingdom?: A systematic review with meta-analysis’, Aladeokin and Haighton^1^ examined the possible relationship between e-cigarette use and initiation of cigarette smoking in the UK. In their meta-analysis of three longitudinal studies^2-4^, the authors found an unusually large pooled odds ratio (OR) of 26.01 (95% CI: 5.35–126.44). On close examination it is revealed that the very large OR is in fact erroneous and has resulted by entering as log odds the published adjusted ORs from the three longitudinal studies in their generic inverse variance meta-analysis^5,6^. Thus, the ORs being pooled were exponentiated values of the ORs from the studies^7^. Correcting this error shows that the OR remains significant (OR=3.86; p<0.00001) (Figure 1) but much lower than the published OR value of 26.01.

It is noteworthy that this amended meta-analysis has a lower pooled OR (3.86) than the meta-analysis of unadjusted ORs by Aladeokin and Haighton (OR=5.55)^1^. This finding may suggest that controlling for confounders can significantly impact on the outcomes of studies examining the relationship between e-cigarette use and initiation of cigarette smoking. A report by McNeill et al.^8^, synthesising evidence investigating e-cigarette use and its association with initiation of cigarette smoking among adolescents in the UK, supports the importance of controlling for variables in e-cigarette studies^8^. The report found that although e-cigarette users were more likely than non-users of e-cigarettes to try cigarette smoking, there was no established evidence to suggest progression to cigarette smoking. In reaching the foregoing conclusion, McNeill et al.^8^ cited two of the three studies used by Aladeokin and Haighton, viz Conner et al.^2^ and Best et al.^3^, and suggested that these studies were challenged by their inability to properly control for all relevant confounders, which limited their capacity to determine causality^8^.

Until further studies are conducted, evidence linking e-cigarette use to cigarette smoking initiation must be interpreted with caution.
